# Improving the early presentation of cancer symptoms in disadvantaged communities: putting local people in control

**DOI:** 10.1038/sj.bjc.6605390

**Published:** 2009-12-03

**Authors:** D Lyon, J Knowles, B Slater, R Kennedy

**Affiliations:** 1 The Improvement Foundation, 57 Spring Gardens, Manchester M2 2BY, UK

**Keywords:** health inequalities, early presentation, improvement, community development, innovation

## Abstract

**Background and aims:**

Premature deaths from cancer affect deprived communities disproportionately. The Department of Health has funded a programme in 19 Spearhead PCTs, delivered by the Improvement Foundation, to promote the early presentation and diagnosis of breast, bowel and lung cancers, with the ultimate intention of improving outcomes.

**Methods:**

The programme uses improvement methodology and is a unique approach involving local people working in partnership in their communities to raise awareness of cancer symptoms and promote early presentation. The teams work with primary care, other statutory organisations and with the voluntary sector. The specific contribution of the local people has been in the identification of hard-to-reach groups and the tailoring of effective health messages.

**Results:**

Interim results show an increase in the number of urgent 2-week referrals and the proportion of new cancer cases diagnosed through the urgent 2-week referral route (from 43% to 51%) for all three cancers. These results were statistically significant for the bowel cancer and lung cancer pathways. There was also an increase in the proportion with no spread at the time of diagnosis for bowel cancer (38–43%) and breast cancer (41–44.5%), but these results did not reach statistical significance.

**Discussion:**

This programme, helping community volunteers to lead work on raising awareness and promotion of earlier presentation of cancer symptoms in partnership with primary care and other professionals, is delivering positive early results.

One- and 5-year survival rate statistics show that people living in the most deprived areas of England are less likely to survive common cancers than those living in more affluent areas (Ellis *et al*, 2009). The reasons for deprived communities having generally poorer health are complex, but well documented, and include effects of unemployment, low income, poor housing and low levels of literacy, often exacerbated by lifestyle choices (Graham, 2007). Those factors relating specifically to health and health services include poor levels of health knowledge, low expectations from statutory services and expectancy of a shortened life and poor health in middle age. Coupled with these are often low self-esteem and lack of confidence when interacting with professionals. In some areas of high deprivation, there is a paucity of good quality, accessible primary health care and in others, little understanding of how to use primary care services to best effect (Mercer and Watt, 2007). Many of these factors not only impact on the onset of particular health problems, but also on the stage at which health problems (particularly cancer) are diagnosed. Therefore, a programme to promote the earlier presentation of cancer symptoms has a particular relevance in deprived communities.

This paper provides interim results from the first year of an improvement programme, commissioned by the Health Inequalities Unit at the Department of Health, to promote earlier presentation of symptoms of bowel, breast and lung cancer in some of the most deprived areas of England. This programme adopts the Improvement Foundation's *Healthy Communities* approach, which has previously been applied to other health topics (Department of Health, 2004; Slater *et al*, 2008).

Only around a third of new cancer cases in the United Kingdom are diagnosed as a result of general practitioner (GP) referrals through the urgent 2-week system (Rosen *et al*, 2006). The other two-thirds are diagnosed through a range of routes, including non-urgent referrals, screening, out-patient hospital appointments or patients presenting as emergencies to hospitals. These other routes are more likely to include late presentations of cancer. The focus of the *Healthy Communities* intervention is, therefore, to provide resources, activities and support that encourage the community to seek medical attention from their GP for early symptoms.

The *Healthy Communities* programme supports community volunteers to work in partnership with primary care staff and other specialist cancer service providers, in both statutory and voluntary sectors, to lead improvement locally. Community members and professionals are taught to use improvement tools to identify what can be changed to make an improvement, and then to measure that improvement. Outcomes include not only improvement in a specific topic area, but benefits to the individual volunteers and to the community itself. The approach is unique in the way it galvanises communities to have a direct impact on health care and health outcomes.

## Materials and method

Ten Spearhead (areas defined as those in the bottom fifth for three out of five selected health indicators) primary care trusts (termed ‘sites’) participated in the first wave of the programme, comprising 111 practices with a combined practice population of 630 000 and >343 whole-time-equivalent GPs. The first wave started reporting in October 2007 and ended in September 2009. A second wave of the programme comprising a further nine sites started in October 2008 and will complete in September 2010. Data from the first wave for the period ending in September 2008 are presented in this paper.

All sites identified three or more teams to work in discrete communities across their area. Each local area team covered a population of 10 000–12 000 and consisted of around 12 individuals, at least half of whom were volunteers. The remaining team members were professionals drawn from the statutory and voluntary sector in the local area. Some professionals, such as specialist cancer nurses, provided support across all three teams, while others were team specific. A project manager appointed by the lead organisation led the teams and coordinated the work at primary care trust level.

### Raising awareness

The make-up of the teams, and particularly the involvement of community members, was hugely beneficial and helped to ensure that awareness-raising messages used to promote earlier presentation were culturally appropriate and delivered in an accessible and engaging format.

Venues and occasions were recognised to be important in shaping the way that messages are delivered and received. When the teams considered the settings where people are usually exposed to information and cancer literature – doctors’ waiting rooms, hospital out-patients’ departments, health clinics and so on – it was clear that there was not much scope to inject humour and openness into the messages. For this reason, the teams found the literature produced nationally of limited value and so developed their own creative messaging and methods of delivery.

One key focus was to dispel myths and preconceptions about cancer by showing that the diagnosis of cancer is not necessarily a death sentence and that if made at an early stage, treatment can lead to cure. The intention was to make cancer no longer something to be confronted in a fearful way and only in a clinical setting.

The teams found ways to take the same messages into a pub, bingo hall or community hall, venues often frequented by the older population in whom cancer is most common, and to shape them into a game or a quiz for their neighbours. This afforded a different perspective and potentially a greater understanding of what was being communicated.

Where minority communities were involved, links into mosques, temples and faith groups were made to understand how best to reach these groups.

Games that facilitated both learning and enjoyment were developed, including a breast cancer ‘snakes and ladders’ game and bowel cancer bingo. Some people's personal experiences were used as a basis for songs, plays and poems. To tackle the embarrassment older people can feel when invited to take part in bowel cancer screening, one community devised, wrote and performed their own play in a variety of settings. In other communities, volunteers in pubs effectively delivered information to men on bowel and lung cancer symptoms. Men can be reluctant to pick up and read leaflets, but in an area in which bowel and lung cancer symptoms were displayed on beer mats, all the mats were taken.

As well as helping to ensure that messages were delivered in an appropriate and engaging way, the involvement of local people in the awareness-raising activities became a story in itself. Local media have been keen to report on events, further enhancing message reach.

### Measurement

**Figure 1:**
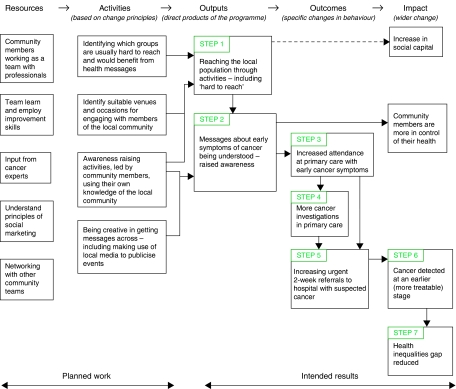
Logic model underpinning the healthy communities programme to encourage the early presentation of cancer.

A simple logic model (McLaughlin and Jordan, 2004) was used to identify measures to best indicate the success of the programme in delivering its intended outcomes. Logic models are a way of showing how programmatic activities are connected to client or consumer outcomes, thereby identifying points of enquiry at which one can reasonably expect to see specific outcomes. They can be used alongside more sophisticated and complex ‘theories of change’ (Blamey and Mackenzie, 2007) methods to provide an evaluation framework for social change interventions. The logic model underpinning the *Healthy Communities* programme delivery is given in Figure 1.

Seven steps were identified in the delivery of the intended impact of the programme:

*Step 1*: *Reaching the local population* – To promote earlier presentation of breast, bowel and lung cancer symptoms, the programme first needs to reach its target population. To assess this, healthy community teams recorded the number of people attending events. However, this captures only a proportion of the ‘reach’ of the programme, which might be conveyed through friends and family and wider media publicity.

*Step 2*: *Raising awareness* – An essential part of the programme is increasing awareness of cancer symptoms. This was not assessed through any formal means in the first wave of the programme.

*Step 3*: *Attendance in primary care* – Given the programme's aim to promote earlier presentation, attendance in primary care, in terms of volume and timeliness, is an important indicator. One way in which to collect these data is through use of Read codes on the general practice computer system (Chisholm, 1990).

*Step 4*: *Investigation in primary care* – On the basis of more people presenting with potential lung cancer symptoms, one might expect GPs to undertake more chest X-rays, but they are less likely to initiate investigations into bowel or breast symptoms. This means that there is no easily collectable investigation measure for the three cancers.

*Step 5*: *Onwards referral to hospital* – The urgent 2-week referral pathway from primary care to hospital is an important indicator of suspect cancer presentations in primary care and is reliably captured in national monitoring statistics. Two-week referrals would be expected to increase if more patients were presenting at primary care with suspected cancer symptoms. Furthermore, by collecting the number of new cases of cancer, the proportion of cases diagnosed through the 2-week route could be calculated. If more people present to primary care early, rather than through emergency routes such as A/E, the proportion of new cancer cases diagnosed through the urgent 2-week referral route would be expected to rise.

*Step 6*: *Earlier stage at diagnosis* – If more cancers are identified through the 2-week referral route, one would expect them to be diagnosed at an earlier stage in the progression of the disease. Direct evidence of this would be provided by recording or application of a valid staging model at diagnosis. Staging is usually undertaken by cancer specialists in secondary care, but is often poorly recorded even in hospitals. General practices tend to receive information on spread of the disease through reports on surgical procedures, and knowledge of disease severity is required by GPs for the purposes of the new cancer patient review undertaken as part of the quality and outcomes framework (QOF) (NHS Information Centre, 2009).

*Step 7*: *Contribution to reduced health inequalities* – The ultimate goal of the improvement programme is to narrow the gap in mortality between the most deprived areas in England and the national average. One-year survival is a potential indicator of this.

*Monthly measures and data collection* Measures from which to drive improvement for steps 1–4 were not practical at the time the programme started, and because of the length of time involved for data collection, a measure for step 7 was not an appropriate improvement-driving measure. The emphasis has, therefore, been put on step 5 (2-week wait referrals) and step 6 (spread of cancer at diagnosis).

Participating GP practices were expected to collect data every month for the duration of the programme and to establish a baseline by looking back over a 12-month period before programme commencement. Data collection was designed to be simple and the monthly measures were used locally to drive practice improvement as well as contribute to the overall evaluation of the programme.

The data collected by participating GP practices were as follows:

1. The number of urgent 2-week referrals for bowel, breast and lung cancer.
2. The number of new bowel, breast and lung cancer cases diagnosed.
3. The proportion of new cancer cases diagnosed through urgent 2-week referral for bowel, breast and lung cancer.
4. The proportion of new cancer cases for bowel and breast with no spread of disease detected at diagnosis.

The GPs were instructed to use a simplified ‘TNM’ classification: no spread, localised spread or distant spread (Greene *et al*, 2004). For breast cancer, no spread is node-negative cancer confined to one breast with no distant spread. For bowel cancer, no spread is Duke's A or Duke's B.

Practices use the Read code system to record consultations, including diagnoses on the electronic patient record. The QOF of the general practice contract rewards practices for the quality of the care they provide, especially for people with long-term conditions. To establish whether practices are carrying out the care that qualifies for the payments, the electronic records are scrutinised for specific Read codes.

New cancer cases have a section within the QOF, so practices are very efficient at entering the relevant Read codes whenever a new diagnosis of cancer is made. This measure was used as a denominator for our measures ‘three’ and ‘four’. The numbers of new cancer cases are small, even in a large practice, so it is a simple task for a clinician to review each case, which is also a requirement of QOF, to establish whether the case was diagnosed after an urgent 2-week referral and whether there was spread at the time of diagnosis. The presence of spread is easier to determine when a surgical procedure has been carried out. Operations are often carried out for bowel and breast, but not for lung cancer. Unfortunately, details of operative findings are often sent to the practices some time after the initial diagnosis.

## Results

### Establishing a baseline

Establishing a baseline number of new cancer cases and whether they were referred through the urgent 2-week system was relatively easy for practices because the numbers were small. A total of 86% of practices managed to collect a baseline and monthly data for a full 12 months for these measures. However, because of the delays in practices receiving information about spread, only 61% managed to collect both the baseline and a full 12 months (see Table 1).

Recording all urgent 2-week referrals is not a requirement of QOF. There are Read codes available, but few practices use them routinely. Once the practices used the codes, it was simple to collect ‘measure one’ prospectively, but only 56% of practices were able to establish a baseline.

These data were validated by scrutinising various hospital systems in which recordings of urgent 2-week referrals, new diagnoses and spread are also made. In addition, estimates of expected numbers were in keeping with public health data. This was established locally for referrals and nationally for new diagnoses and spread. The baseline for bowel cancer with no spread at 38% is comparable with the national percentage of bowel cancer with Duke's grading of A or B. For breast cancer, the baseline finding of 41% with no spread is in keeping with the national figure of node-free disease at the time of operation.

### Reported outcomes at the end of year one

The results reported are interim results at the end of year one of the 2-year programme for the first wave of 10 sites. As interim results, they are only indicative of progress to date. Table 1 presents each of the outcome measures as a comparison between baseline and year one. Data are included only for those practices that were able to supply both a baseline and monthly measures for any particular item.

**Figure 2:**
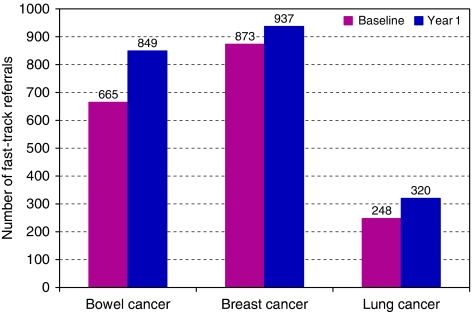
Urgent 2-week referrals for bowel, breast and lung cancer (outcome measure 1).

**Figure 3:**
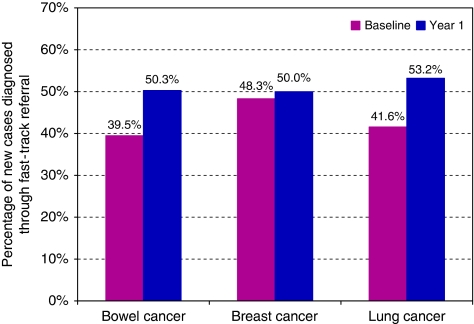
Percentage of new cancer cases diagnosed through urgent 2-week referral route (outcome measure 3).

**Figure 4:**
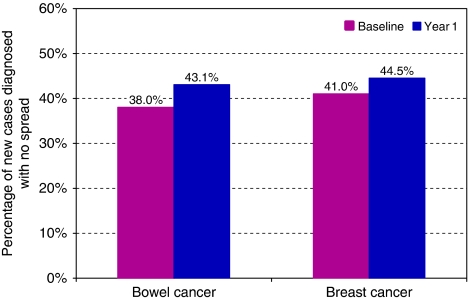
Percentage of new cancer cases diagnosed with no spread (outcome measure 4).

Figures 2, 3 and 4 show a comparison between baseline and year one for each of the three outcome measures. Figure 2 illustrates the interim results for the number of urgent 2-week referrals for bowel, breast and lung cancer for the 63 (57%) practices supplying complete data. For those that could not, the main reason was difficulty in collecting data retrospectively. The results show an increase in urgent 2-week referrals for all cancer sites. Both suspected bowel cancer and suspected lung cancer show increases of over 25%. The increase in suspected bowel cancer is statistically significant (*χ*^2^=22.193, df=1, *P*<0.0001) and the increase in suspected lung cancer is also statistically significant (*χ*^2^=8.886, df=1, *P*=0.003). Suspected breast cancer urgent referrals also increased, but the increase is not statistically significant (*χ*^2^=2.204, df=1, *P*=0.138).

Ninety-five of the participating practices (86%) supplied data indicating the proportion of new cancer cases diagnosed through the urgent 2-week pathway. Figure 3 shows that at the end of year one, diagnoses of cancer by the urgent 2-week route accounted for at least 50% of new cancer diagnoses for each of the three cancers. The overall proportion of cancers diagnosed through the urgent 2-week route for all three cancers increased from 43% to 51%. The increase in proportion of bowel cancers detected through the urgent 2-week route is statistically significant (*χ*^2^=4.687, df=1, *P*=0.03). The increase in proportion of lung cancers detected through the urgent 2-week route is statistically significant (*χ*^2^=9.178, df=1, *P*=0.002). An improvement in the proportion of breast cancers detected through the urgent 2-week route was also observed, but this is not statistically significant (*χ*^2^=0.106, df=1, *P*=0.745).

Sixty-eight practices (61%) supplied data on spread of the cancer at diagnosis for baseline and year one (Figure 4). The practices found it relatively easy to consistently collect this measure for breast and bowel cancer. However, there was difficulty in defining and reporting on spread for lung cancer because most patients do not undergo surgery and are, therefore, not pathologically staged. Consequently, this paper does not present data for lung cancer, although these data are available on request. The proportion of cases of bowel cancer with no spread at diagnosis seemed to increase, but this did not reach statistical significance (*χ*^2^=0.93, df=1, *P*=0.34). Similarly for breast cancer, the proportion of cases with no spread at diagnosis showed an increase that was not statistically significant (*χ*^2^=0.633, df=1, *P*=0.426).

## Discussion

### Statement of main findings

A programme that helps community volunteers to lead work on raising awareness and promotion of earlier presentation of cancer symptoms in partnership with primary care and other professionals is delivering positive early results. The improvements in detection of bowel cancer and lung cancer, both in terms of the number of urgent 2-week referrals, and the proportion of new diagnoses coming through the urgent 2-week route have been shown to be statistically significant. There have been improvements in the detection of breast cancer, and in the extent of spread of bowel and breast cancers at diagnosis, but these are not statistically significant. It seems that for bowel and lung cancers, the *Healthy Communities* programme may have encouraged people to present sooner with their symptoms and this has impacted on the health system diagnostic route. The failure of improvements in the breast cancer diagnostic pathway to reach significance may be because a higher media profile over a long period of time has increased familiarity with the symptoms of early breast cancer in these communities and that these symptoms are also easier to describe and to spot. Although there have been improvements in the proportion of cancers diagnosed with no spread at diagnosis, these changes are not statistically significant after 1 year.

It is not possible to say with certainty that the programme activities are directly responsible for the improvements reported in this paper, but we are not aware of any other activity, taking place at the time, that would have caused these outcomes. The strengths of the programme are the involvement of community members and the creativity that members show, the flexibility of approach (that may not have been considered without local involvement) and the opportunity to link community involvement to clinical professional expertise.

### Limitations of the study

This programme is a social intervention for a problem that has social roots and social characteristics, but is linked to the measurement of hard outcomes. This paper presents interim results obtained after 1 year of a 2-year programme. Limitations include the lack of comparative data from practices not exposed to a *Healthy Communities* intervention, the lack of assessment of awareness levels in the general or target population, and missing data for some of the practices involved. In particular, some general practices were not able to access baseline data for all the measures, the use of Read codes was inconsistent and there was often a delay in practices obtaining complete and accurate information about spread of disease at diagnosis, especially for lung cancer.

It should also be acknowledged that the programmes were undertaken within some of the most disadvantaged communities in England. This, and the idiosyncratic nature of activities undertaken across the sites, may limit the extent to which the results can be generalised. However, this work addresses the needs of people who may often be described by professionals as ‘hard to reach’. Hence, it is of interest to both policy makers and professionals and, as awareness increases, to the communities themselves.

This programme has been delivered using an innovative approach developed by the Improvement Foundation that drives improvement through engagement of community members and clinical professionals. Early indications are that putting lay people in a lead role to improve health and wellbeing in disadvantaged communities is producing very promising results.

## Conflict of interest

The authors declare no conflict of interest.

## Figures and Tables

**Table 1:** Comparison of year 1 with baseline for main outcome measures

**Outcome measure**		**Cancer site**	**Practices number (as % of participating practices)**	**Practice population coverage**	**Baseline**	**Year 1**	**Percentage improvement**	***χ*^2^ statistic (with Yates’ correction)**	***P*-value**	**Statistical significance**
1	Number of urgent 2-week referrals	Bowel	63 (57%)	229 400	665	849	27.7	*χ*^2^=22.193, df=1	*P*<0.001	Highly significant
		Breast	63 (57%)	229 400	873	937	7.3	*χ*^2^=2.204, df=1	*P*=0.138	Not significant
		Lung	63 (57%)	229 400	248	320	29.2	*χ*^2^=8.886, df=1	*P*=0.003	Highly significant
3	Percentage of new cases diagnosed through urgent 2-week referral	Bowel	95 (86%)	394 200	39.5% (90/299)	50.3% (98/195)	27.4	*χ*^2^=4.687, df=1	*P*=0.03	Significant
		Breast	95 (86%)	394 200	48.3% (144/298)	50.0% (146/292)	3.5	*χ*^2^=0.106, df=1	*P*=0.745	Not significant
		Lung	95 (86%)	394 200	41.6% (160/225)	53.2% (175/329)	28.0	*χ*^2^=9.178, df=1	*P*=0.002	Highly significant
4	Percentage of new cases with no spread at diagnosis	Bowel Breast	68 (61%) 68 (61%)	255 700 255 700	38.0% (87/229) 41.0% (122/298)	43.1% (98/195) 44.5% (130/292)	13.5 8.5	*χ*^2^=0.93, df=1 *χ*^2^=0.633, df=1	*P*=0.34 *P*=0.426	Not significant Not significant
